# Influencing factors of low-dose computed tomography screening behavior among first-degree relatives of lung cancer patients based on the theory of planned behavior: a qualitative study

**DOI:** 10.3389/fpubh.2026.1896783

**Published:** 2026-08-21

**Authors:** Si Lin, Yaming He, Weixuan Ye, Xiaokun Wu, Lixue Liang, Yinsong Chen

**Affiliations:** 1 The Eighth Clinical Medical College of Guangzhou University of Chinese Medicine, Foshan, China; 2 Department of General Surgery, Foshan Hospital of Traditional Chinese Medicine, Foshan, China; 3 Department of Gastroenterology, Foshan Hospital of Traditional Chinese Medicine, Foshan, China; 4 Department of Cancer Center, Foshan Hospital of Traditional Chinese Medicine, Foshan, China

**Keywords:** first-degree relatives, lung cancer, qualitative research, screening behavior, theory of planned behavior

## Abstract

**Objective:**

To explore the factors influencing the screening behavior of first-degree relatives of lung cancer patients toward low-dose computed tomography (LDCT) screening based on the Theory of Planned Behavior (TPB), gain deeper insights into their subjective experiences and core demands, and provide a theoretical basis and improvement measures for enhancing screening participation rates among this high-risk population.

**Methods:**

Employing a descriptive phenomenological approach, semi-structured interviews were conducted with 14 first-degree relatives of lung cancer patients at a hospital from May to July 2025. Thematic analysis was performed using directed content analysis.

**Results:**

Guided by the TPB, 11 themes across 3 dimensions were identified: Behavioral Attitudes (affirming screening value, family environment influence, cognitive biases hindering screening), Subjective Norms (influence of others’ behavior, healthcare professional recommendations, rural cognitive limitations), and Perceived Behavioral Control (financial constraints, work-time conflicts, cumbersome screening procedures, negative psychological perceptions, policy reference effects).

**Conclusion:**

Screening behaviors among first-degree relatives of lung cancer patients are influenced by multidimensional factors. Healthcare providers should integrate the TPB framework and implement multi-level improvement measures—including enhanced health education, optimized screening services, and improved policy support—to increase screening adherence.

## 1 Introduction

According to the Global Cancer Statistics Report, approximately 2.5 million new lung cancer cases were diagnosed worldwide in 2022, accounting for 12.4% of all new cancer cases and ranking as the most prevalent malignant tumor globally (1). In 2022, the number of new cases and deaths of lung cancer in China reached 1.06 million and 733,000, respectively. Its incidence and mortality rates continued to rank first among all types of malignant tumors, posing a serious threat to public health (2). Epidemiological studies indicate that genetic factors interact with environmental exposures, lifestyle, and other risk factors, leading to a significantly increased risk of lung cancer among first-degree relatives-specifically parents, children, and siblings-of lung cancer patients (3). A study on lung cancer screening found that the detection rate of lung cancer reached 6.2% among high-risk individuals under the age of 50 with a family history of lung cancer in first-degree relatives (4). Tumor-Node-Metastasis (TNM) staging is closely associated with the prognosis of lung cancer patients; the 5-year overall survival rate for patients with stage IA1 is as high as 82%, while that for patients with stage IVB is only 7% (5). Multiple studies have demonstrated that early screening using low-dose computed tomography (LDCT) significantly improves survival rates among lung cancer patients (6–8). The 2020 National Comprehensive Cancer Network guidelines for lung cancer screening explicitly state that individuals aged ≥50 years with a smoking history of ≥20 pack-years and a family history of lung cancer among first-degree relatives should be prioritized for lung cancer screening (9). The Chinese Guidelines for Lung Cancer Screening and Early Diagnosis and Treatment (2021, Beijing) also emphasize that early screening for first-degree relatives of lung cancer patients holds significant clinical value, recommending LDCT as the screening method (10). However, both clinical efficacy trials and screening programs have reported consistently low participation rates for lung cancer screening among high-risk populations (11, 12). An analysis of influencing factors indicated that the actual compliance rate for lung cancer screening among first-degree relatives of lung cancer patients, a specific high-risk group, was only 23.9%, lower than the 35.32% participation rate of high-risk individuals who met screening criteria after systematic evaluation in the China Urban Cancer Early Diagnosis and Treatment Program. This difference highlights the unique barriers to screening behavior among high-risk individuals with a clear family history (13). Although studies have explored the influencing factors of lung cancer screening, in-depth analysis of screening behavior among first-degree relatives of lung cancer patients is still lacking (13). Compared to the general high-risk population, first-degree relatives often remain asymptomatic in the early stages of the disease, resulting in generally weaker screening motivation. They frequently decline screening based on the belief that “no symptoms mean no need,” and lack effective guidance from healthcare professionals. Some individuals actively avoid screening due to fatalistic beliefs, fear of positive results, and financial burdens associated with out-of-pocket screening costs (14, 15). Therefore, in-depth research grounded in theoretical frameworks is needed to systematically identify core barriers affecting screening behavior among this population, thereby informing the development of targeted intervention strategies.

**Figure 1:**
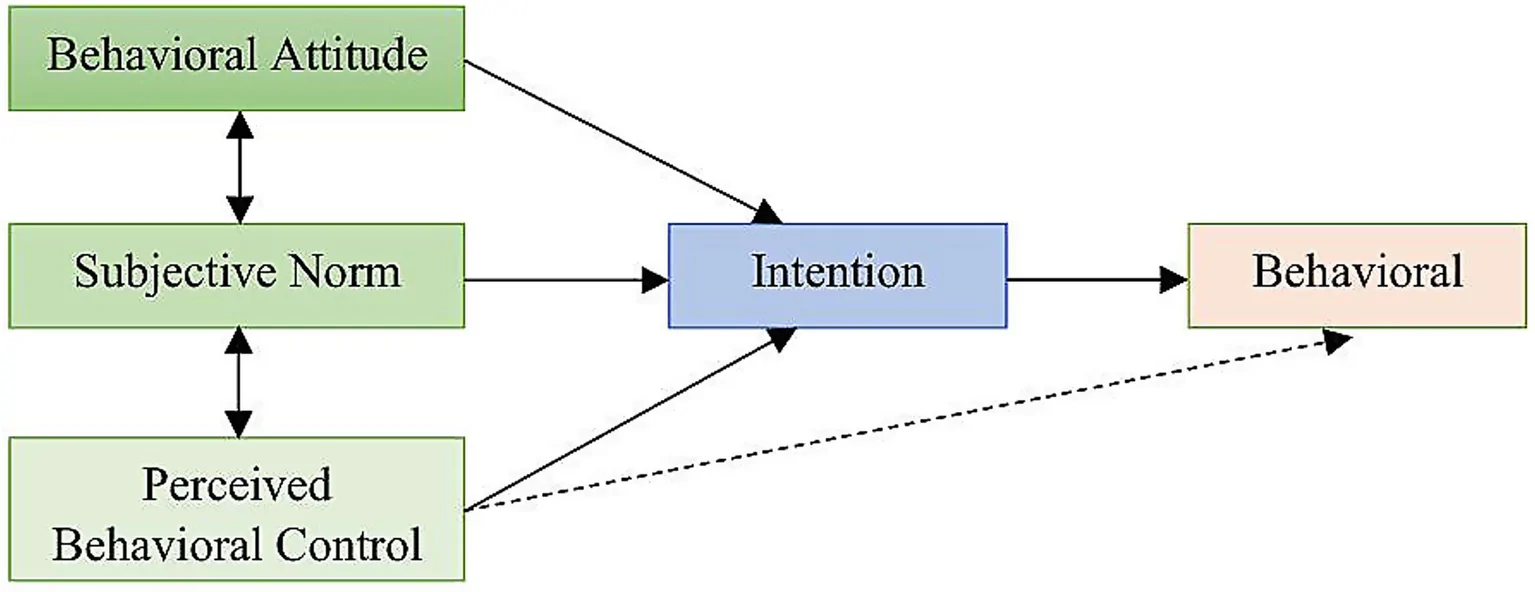
The model of Theory of Planned Behavior (TPB).

The Theory of Planned Behavior (TPB) was proposed by Ajzen (16). As shown in Figure 1, this theory posits that an individual’s behavioral intention is a key determinant of actual behavior, and the formation of this intention is influenced by the synergistic effects of three core dimensions: first is behavioral attitude, which refers to an individual’s positive or negative evaluation of performing the target behavior, based on their value judgment of the behavior’s outcomes; second is subjective norm, reflecting the perceived expectations and social pressure from significant others (e.g., family, physicians) regarding the performance of a specific behavior. Third is perceived behavioral control, which refers to an individual’s assessment of their own ability and resource conditions to perform the behavior. Perceived behavioral control not only indirectly influences behavior through behavioral intention but can also directly impact actual behavior. When individuals hold a positive attitude toward a behavior, perceive strong social support, and believe they have the capability to complete it, their behavioral intention is significantly enhanced. Current research on lung cancer screening behavior primarily relies on quantitative methods, with limited in-depth exploration of the screening population’s authentic experiences, intrinsic motivations, and underlying reasons behind behavioral decisions. Based on the TPB framework and employing qualitative research methods, this study focuses on exploring the factors influencing first-degree relatives of lung cancer patients to undergo LDCT screening. It also aims to understand these relatives’ assumed attitudes toward LDCT screening, their perceived barriers, and their willingness to undergo future screening. The study seeks to provide a theoretical basis for developing scientific and effective early lung cancer screening intervention strategies for this high-risk population, thereby increasing their participation rate in LDCT screening.

## 2 Methods

### 2.1 Design

Based on the TPB framework, this study employs a descriptive phenomenological approach. Through semi-structured in-depth interviews with research subjects, it thoroughly analyzes the factors influencing and the authentic experiences of first-degree relatives of lung cancer patients regarding LDCT screening.

### 2.2 Participants

This study employed purposive sampling to select first-degree relatives of hospitalized lung cancer patients in the Oncology Department of a hospital as research subjects for interviews conducted between May and July 2025. Inclusion criteria: (1) First-degree relatives of lung cancer patients aged ≥18 years; (2) Volunteered to participate in this study and have signed the written informed consent form; (3) Normal cognitive function and adequate communication and comprehension abilities. Exclusion criteria: (1) Individuals with severe cognitive impairment or a history of psychiatric disorders; (2) Individuals with other serious organic diseases; (3) Individuals who withdrew from the study due to uncontrollable factors. The sample size was determined based on the principle of information saturation, meaning recruitment ceased when no new research themes or perspectives emerged during data collection and analysis. Ethical approval for this study was obtained from the local hospital ethics committee.

### 2.3 Develop an interview outline

Researchers developed an interview outline based on the TPB framework. Following a literature review, two oncology nursing experts were invited to review the outline. Pre-interviews were conducted with two first-degree relatives of lung cancer patients. Based on feedback, the outline was further revised and finalized. Interviews employed open-ended questions such as “Could you share your experience when your family member was diagnosed with lung cancer?” and “Are you aware of lung cancer screening? Through which channels do you typically obtain related information?” The detailed interview outline is presented in Table 1.

**Table 1:** Interview outline.

Theoretical framework	Questions
Behavioral attitude	1. What benefits and risks do you think lung cancer screening might bring?
	2. What risks or inconveniences do you think screening might cause you?
Subjective norm	3. Have your family, friends, or doctor recommended lung cancer screening to you? How did their opinions influence you?
	4. Have any of your friends or colleagues undergone lung cancer screening? How did their experiences change your perspective on lung cancer screening?
	5. What is the attitude toward lung cancer screening in your community or cultural environment? How does this influence you?
Perceived behavioral control	6. What factors motivate you to undergo lung cancer screening?
	7. What factors deter you from undergoing lung cancer screening?
	8. Regarding the deterrents mentioned above, what information or assistance would you like to receive to encourage you to undergo screening?

### 2.4 Data collection

This study collected data through one-on-one, face-to-face, semi-structured interviews. The interviews were conducted by a graduate student in nursing who had received training in systematic qualitative research methods but was not directly involved in the clinical care of patients or their families. Prior to the interviews, the interviewer had no prior relationship with any of the participants. At the beginning of each interview, the interviewer clearly informed the participant that she was a “research interviewer” rather than a “nursing staff member” to prevent participants from mistakenly believing that the interview was part of clinical care. Interviews were conducted in a quiet, private office to ensure an undisturbed environment. Before the formal interview, the interviewer explained the purpose and significance of the study in detail to the participant and activated the recording device after the participant signed a written informed consent form. During the interview, the interviewer maintained a neutral and open attitude throughout, encouraging participants to express their genuine thoughts without judging any responses. The interviewer also used techniques such as probing, paraphrasing, and emotional responses to facilitate in-depth communication while strictly avoiding leading questions. The interviewer proactively reminded participants that there were no “right” or “wrong” answers in the interview and that the sole aim was to understand their genuine perspectives. In addition to verbal content, the interviewer observed and recorded nonverbal cues, such as facial expressions, body language, and emotional reactions. This study employed qualitative research methods, and the sample size was determined based on the criteria of data saturation—that is, recruitment was halted when no new themes (codes) emerged or when further coding was no longer possible. Based on the age, gender, educational background, and place of residence of first-degree relatives, and in accordance with the principle of maximum differentiation, when no new themes emerged by the 12th interviewee, and adding two more interviewees still yielded no new themes—while the existing themes were sufficient to fully explain the participants’ experiences—it was determined that the data had reached saturation, and the interviews were immediately terminated.

### 2.5 Data analysis

Data organization and analysis proceeded concurrently. This study employed directed content analysis. Directed content analysis is commonly used when relevant theories and literature on a phenomenon already exist, with the aim of validating or expanding a particular conceptual framework or theory. This method employs a top-down approach to analyze textual data, using existing theories to guide the coding and classification of the data, thereby facilitating a process that moves from theory to data and from the abstract to the concrete. The specific reasons for selecting directed content analysis in this study include: First, the TPB provides a clear theoretical framework for the research. TPB explicitly states that behavioral intention is influenced by the synergistic effects of three core dimensions: behavioral attitude, subjective norm, and perceived behavioral control. This theoretical framework helps us focus on the key factors influencing screening behavior, thereby avoiding haphazard research design and the collection of scattered, disorganized data. Second, by classifying the text using predetermined coding categories, researchers can assess whether the data supports the theoretical assumptions of TPB, thereby validating the theory. Third, directed content analysis typically involves clear theoretical guidance and a predefined coding scheme, making the analysis process more structured. Although this method starts from a predefined theoretical framework, it also allows researchers to assign new codes to texts that cannot be categorized under the predefined categories, which may ultimately lead to the formation of new categories and the expansion of existing theory. This characteristic makes it more suitable for this study than traditional content analysis. The aim is to systematically analyze influencing factors under the guidance of the TPB theory, while not completely excluding important findings that lie outside the scope of the TPB. Two researchers repeatedly listening to recordings and transcribing transcripts verbatim within 24 h after each interview. The transcripts were then returned to participants for verification. All 14 participants confirmed the accuracy of the transcribed content, ensuring data integrity and precision. Interview transcripts were systematically analyzed using NVivo 15.0 software. When using directed content analysis, the following steps are included: (1) Constructing initial coding categories based on the framework of the TBP; (2) By repeatedly reading word by word, focusing on content related to the research question or theory, marking and annotating it, and extracting semantic units; (3) Classifying the relevant semantic units using predetermined coding categories and creating codes; (4) After completing all coding, the researcher classifies and compares the codes, merges codes with similar meanings and verifies their rationality, and also determines whether subcategories need to be constructed based on the similarities and differences between codes. Texts that cannot be classified using the initial coding scheme are assigned new codes, which may eventually form new categories and expand existing theories; (5) analyzing each category in depth, refining the core themes, and constructing a conceptual framework; and (6) returning to the original text to check the appropriateness of the themes and making necessary adjustments. To ensure research validity, the researcher effectively controlled subjective bias through methods such as writing reflective journals and organizing coding discussions, ensuring that research findings authentically reflected the interviewees’ perspectives.

To ensure no important topics were overlooked within the TPB framework, researchers adopted the following strategies throughout the analysis to maintain an openness to topics outside the TPB framework. First, an open attitude during the initial coding phase. Although researchers established initial coding categories based on the three dimensions of TPB (behavioral attitude, subjective norms, and perceived behavioral control), they marked and annotated all content relevant to the research question while reading the text word by word. They paid attention not only to content consistent with TPB theory but also to expressions that could not be immediately categorized into the pre-set categories. Second, for texts that could not be classified using the initial coding scheme, researchers did not force them into the TPB framework but marked them as “other,” subsequently conducting independent review and classification. This step ensured that important topics not entirely aligned with TPB were identified and retained. Third, regular discussions and reflections within the research team. The research team held regular discussion meetings during the coding process, reviewing not only the consistency and accuracy of the coding but also discussing whether there was meaningful text content that could not be categorized into existing categories. Researchers record their preconceived notions about the TPB framework by writing reflective journals and consciously examine whether important topics have been overlooked due to theoretical assumptions. Fourth, following the steps of directed content analysis, after completing all coding, researchers determine whether subcategories or new categories need to be constructed based on the similarities and differences between the codes. When multiple codes have similar meanings and collectively point to a new concept that goes beyond the preconceived framework of TPB, researchers will aggregate them into subcategories or new categories.

If the authors encounter disagreements regarding coding, they will resolve them in the following steps: (1) Two researchers independently complete the full text coding and cross-check to extract the items in dispute; (2) The two researchers compare the original interview records and negotiate the interpretation of each item; (3) If the negotiation fails, the research team (including professors with extensive experience in qualitative research) will discuss and decide on the matter collectively; (4) All disputed content will be fully recorded in a reflection log; (5) The final theme summary will be returned to the interviewee for verification and correction to ensure that the interpretation is consistent with the interviewee’s true feelings.

This study was also reported according to the Consolidated Standards for Reporting Qualitative Research (COREQ) guidelines (17), as detailed in Appendix Table 1.

### 2.6 Quality control

Prior to interviews, they proactively inquired about the physical well-being of first-degree relatives to establish rapport. During formal interviews, researchers maintained value neutrality and set aside personal assumptions. Employing active listening and flexible follow-up questions based on a semi-structured interview guide, they comprehensively gathered verbal and nonverbal information from participants. Transcribed transcripts were returned to interviewees for verification and confirmation. During data analysis, the two researchers independently conducted coding and ensured consistency through mutual cross-checking. Throughout the process, they continuously reflected on their personal perspectives, repeatedly reviewed transcripts, and deeply contemplated the implications of their codes. Disagreements over coding or thematic interpretations were resolved through collective team discussions to reach consensus. The entire research process-including data collection, organization, and analysis-was conducted under the guidance of an experienced qualitative research professor. All participants received systematic training in qualitative research methodologies. The study adhered to rigorous protocols, implementing comprehensive quality control measures from data collection through analysis to ensure the authenticity and reliability of findings.

## 3 Results

This study interviewed 14 first-degree relatives of lung cancer patients (coded N1–N14), with interview durations ranging from 21 to 33 min (mean = 26.5 min). The mean age of participants was 38.93 ± 10.01 years. The sample comprised 10 women and 4 men. Among them, 3 participants (all male) had a history of smoking, with pack-year values of 8, 9, and 52.5, respectively. Nine participants (64.3%) reported a history of secondhand smoke exposure, and one participant (N11, a chef) reported occupational exposure to dust, asbestos, chemical fumes, or other known pulmonary carcinogens. In terms of marital status, 11 were married and 3 were unmarried. Educational levels ranged from junior high school to undergraduate degrees. Regarding residence, 9 participants lived in urban areas and 5 in rural areas. The majority (12/14, 85.7%) had a monthly household income exceeding 3,000 yuan. Health insurance coverage included basic employee medical insurance (*n* = 5), resident medical insurance (*n* = 6), and the New Rural Cooperative Medical System (NCMS, *n* = 3). Only one participant (N11) had a history of LDCT screening. Detailed demographic and clinical characteristics are presented in Table 2. This study identified 3 categories and 11 themes, as shown in Table 3.

**Table 2:** General characteristics of study participants (*n* = 14).

Number	Age	Gender	Smoking or not (number of packs smoked per day × number of years smoked)	History of secondhand smoke exposure	Relationship with patients	Marital status	Education level	Occupation/occupational exposures	Place of residence	Average monthly household income	Type of health insurance	Medical history	History of LDCT screening
N1	35	Women	No	Yes	Father and daughter	Married	U	Company employee/NO	City	3,000–4,000	Employee medical insurance	No	No
N2	44	Men	No	Yes	Mother and son	Married	J	Company employee/NO	City	>5,000	Employee medical insurance	No	No
N3	43	Women	No	Yes	Father and daughter	Married	T	Technical professional/NO	City	>5,000	Resident medical insurance	No	No
N4	22	Women	No	No	Mother and daughter	Unmarried	U	Technical professional/NO	City	3,000–4,000	Resident medical insurance	No	No
N5	53	Women	No	No	Mother and daughter	Married	H	Retire/NO	City	>5,000	Employee medical insurance	No	No
N6	40	Women	No	No	Mother and daughter	Married	T	Technical professional/NO	City	3,000–4,000	Resident medical insurance	No	No
N7	40	Women	No	Yes	Father and daughter	Married	J	Homemaker/NO	Countryside	1,000–2000	NCMS	No	No
N8	36	Women	No	Yes	Father and daughter	Married	U	Technical professional/NO	City	>5,000	Employee medical insurance	No	No
N9	42	Women	No	No	Mother and daughter	Married	M	Homemaker /NO	Countryside	1,000–2000	NCMS	No	No
N10	27	Men	Yes/9 pack-years	Yes	Father and son	Unmarried	T	Technical professional/NO	City	3,000–4,000	Resident medical insurance	No	No
N11	55	Women	No	No	Mother and daughter	Married	M	Chef/Yes	City	3,000–4,000	Resident medical insurance	No	Yes
N12	24	Women	No	Yes	Mother and daughter	Unmarried	J	Freelancer/NO	Countryside	3,000–4,000	Resident medical insurance	No	No
N13	49	Men	Yes/52.5 pack-years	Yes	Father and son	Married	J	Technical professional/NO	Countryside	3,000–4,000	Employee medical insurance	No	No
N14	35	Men	Yes/8 pack-years	Yes	Father and son	Married	M	Freelancer/NO	Countryside	3,000–4,000	NCMS	No	No

M, middle school; H, high school; T, technical secondary school; J, junior college; U, undergraduate; NCMS, new rural cooperative medical system; LDCT, low-dose computed tomography.

Occupational exposures refers to self-reported long-term contact with dust, asbestos, chemical fumes, or other known pulmonary carcinogens.

The history of LDCT screening includes any previous low-dose CT screening for any reason, whether a routine health check-up or symptom-driven.

**Table 3:** Summary of identified categories and themes.

Categories	Themes
Behavioral attitude	Affirming the value of screening Influenced by family environment Cognitive biases impede screening behavior
Subjective norm	Influence of other people’s behavior Recommendations from healthcare professionals Limited awareness in rural areas
Perceived behavioral control	Economic stress restrictions Work schedule conflicts Complex screening processes Negative psychological perceptions Policy reference effects

### 3.1 Behavioral attitude

Attitudinal behavior refers to the degree of cognitive preference toward screening behavior among first-degree relatives of lung cancer patients, representing subjective evaluations and perceptions of screening behavior based on personal values (16).

#### 3.1.1 Affirming the value of screening

Most first-degree relatives hold a positive attitude toward lung cancer screening, believing that early detection can improve cure rates, reduce treatment costs in advanced stages, alleviate anxiety and fear, and enhance family quality of life.

N5: “I think the greatest benefit of screening is detecting potential issues early. Lung cancer often has no symptoms in its early stages, and by the time symptoms appear, it’s usually already in the middle or late stages, significantly lowering the cure rate. Screening helps us catch it when it’s still small and easier to treat. Plus, a normal result gives me peace of mind and reduces unnecessary anxiety.”

N9: “Early detection and treatment are definitely key. Small problems untreated become big ones, and big problems untreated cost lives. Look at those who get screened early-they spend less, suffer less, and recover faster. But if you tough it out and wait until it’s severe, not only does your body suffer, but money flows out like water, and in the end, it might not even be curable.”

N10: “The biggest benefit is early detection. With late-stage cases like my father’s, both the difficulty and cost of treatment increase exponentially. If screening could catch it early, the cure rate would be much higher, and the pressure on our family would not be so overwhelming.”

#### 3.1.2 Influenced by family environment

Family environment influences attitudes toward screening in multiple ways. First, the transmission of health concepts among family members directly impacts individual perceptions of screening. Respondents with relatives in medical fields often exhibit more positive attitudes toward screening.

N8: “My husband suggested I should get screened for my condition-he’s a surgeon at this hospital.”

Family responsibility awareness exhibits gender differences, with female respondents more likely to view screening as part of their capacity to care for the household.

N11: “After all, at my age, I have older adult parents and young children to look after—so many responsibilities. If I were to fall ill myself, what would become of this family?”

Family medical history significantly strengthens household screening awareness, with households reporting a family history of lung cancer exhibiting more proactive screening behaviors.

N4: “My uncle passed away from lung cancer at 39. My maternal grandfather was diagnosed with lung cancer around my mother’s age. My mother also mentioned that my maternal grandmother died from the same cancer. So genetic predisposition likely plays a role in our family. Screening is crucial for us, and I pay extra attention to my own health and that of my loved ones.”

#### 3.1.3 Cognitive biases impede screening behavior

Due to the influence of traditional beliefs and educational levels, some first-degree relatives hold misconceptions about lung cancer screening, failing to recognize its importance. These erroneous perceptions lead to reluctance to undergo screening. Respondents generally underestimate familial genetic risk; despite knowing the patient’s medical history, they still view lung cancer as an isolated event.

N13: “There’s no history of this disease in our family. My dad was the first to get it, so I do not think it’s hereditary. His illness definitely came from smoking for so many years and his lifestyle habits. Older generations were used to being frugal and did not prioritize health maintenance, so it was caused by multiple factors. I do not believe his illness has any familial inheritance. Many people smoke, but that does not mean smoking will cause this disease.”

N6: “I worry about heredity too, but I heard that for those over 70, it’s mostly environmental factors-genetic influence is lower, right?”

Additionally, some first-degree relatives lack accurate understanding of lung cancer screening, believing it does not necessarily lead to early detection. Thus, they see no need for screening. N3: “Why should I get screened? I do not see the benefit-it just scares me. I doubt it guarantees early detection and treatment. Some cases might not even show up on tests. Even if you have a genetic predisposition, it does not mean you’ll definitely get it.”

Some respondents admitted fearing the results and facing reality: N12: “I’m worried about the outcome-scared I might have it. Then I’d be terrified of how to cope, wondering if I’m really going to die. Dying so young? I’d be genuinely anxious before getting tested.”

### 3.2 Subjective norm

Subjective norms typically refer to the mechanism by which individuals perceive social pressure and the influence of others’ expectations on their behavioral intentions during decision-making processes (16).

#### 3.2.1 Influence of other people’s behavior

In decision-making regarding lung cancer screening, the influence of others’ behavior is particularly prominent. Respondents commonly assess the appropriateness of their actions by observing and referencing the screening experiences of people in their immediate social circles.

N3: “I have heard from friends about cancer gene testing. Some friends around me say this is a cellular gene test specifically for cancer.”

N4: “When my mom went in for surgery, the doctor happened to be the same one who had treated my grandfather. My grandfather also passed away from lung cancer. The doctor then mentioned we have a family history of it and advised our family to get regular screenings.”

N8: “My colleague’s father also had lung cancer, so I heard her mention getting this screening. We talked about the treatment back then, but unfortunately, her father was diagnosed too late and was already in critical condition. It’s truly heartbreaking.”

#### 3.2.2 Recommendations from healthcare professionals

Healthcare professionals play a crucial role in conveying health information, and their screening recommendations are a key factor influencing first-degree relatives of lung cancer patients to undergo early detection.

N1: “The doctors never mentioned it. I actually think doctors should emphasize this more. In cases like ours, it really needs to be taken seriously.”

N11: “Our doctors recommended regular check-ups, so after my mother got sick, all three of us siblings went for comprehensive physicals. The results were all normal.”

N14: “During the initial examination at the first hospital, the doctor there suggested our family undergo screening. But after the rushed transfer, we focused solely on the patient and did not have time to think about ourselves. Plus, the doctors at the new hospital did not recommend it either. If the doctors did not mention it, we definitely would not have gone for it.”

#### 3.2.3 Limited awareness in rural areas

Health awareness among rural residents is relatively low, and they are also constrained by economic factors. Most people believe that if they have no symptoms, their bodies are fine and they do not need to get checked.

N9: “I’ve never heard of it before. Today is the first time I’ve heard this term. Rural areas generally have a weaker awareness of these things-people just aren’t very educated.”

N12: “They do not really prioritize it. It feels like people in the countryside nowadays just do not care much-they think it’s a waste of money. Many people feel they do not need checkups if they have no symptoms.

N14: “I think most people just do not pay much attention to it. Rural folks aren’t well-educated and do not understand these things. Plus, they probably think physical exams cost a lot of money and aren’t willing to spend it.”

Moreover, rural areas face limited access to information and experience delays in information dissemination. N13: “It’s just that where we are from is a small place, and many people do not understand these things. Unlike urban folks-they know a lot more.”

### 3.3 Perceptual behavior control

Perceived behavioral control reflects an individual’s past experiences and anticipated barriers (16). When patients perceive they possess more resources and opportunities while anticipating fewer obstacles, their likelihood of engaging in the behavior increases. External factors influencing first-degree relatives’ screening behavior for lung cancer patients primarily include financial constraints, demand for convenient services, negative psychological perceptions, and policy reference effects.

#### 3.3.1 Economic stress restrictions

Economic pressure is the primary external factor influencing lung cancer screening behavior, manifesting mainly in two aspects: the burden of medical costs and concerns about potential treatment expenses. This dual economic pressure leads some high-risk groups to delay or avoid screening.

N1: “If screening were covered by medical insurance or offered through free public programs, I’d be more decisive about getting tested. After all, for ordinary families, out-of-pocket medical expenses are something we have to consider.”

N11: “For us ordinary workers, a single screening costs a lot. If something is found, the follow-up treatment could be a bottomless pit.”

N13: “Free policies would definitely be best. Accompanying my dad for a full physical cost over 2,000 yuan-that’s a significant expense. For ordinary families like ours, it’s a bit of a strain.”

#### 3.3.2 Work schedule conflicts

Work demands significantly impact first-degree relatives’ lung cancer screening behavior, making it difficult for them to prioritize their own health management due to family caregiving responsibilities, occupational stress, and social factors.

N1: “For me, the biggest obstacle is likely scheduling. It’s hard to take time off work now, and many hospitals require CT scans to be booked well in advance. Plus, you have to make multiple trips to the hospital during weekdays.”

N4: “The main issue is work conflicts. Taking time off for the screening is necessary, but the company has been exceptionally busy lately, so it keeps getting postponed.”

N8: “I’m certain work would delay it. First, you need to schedule it on a weekday, then make the appointment. After booking, you still have to wait in line for quite a while-like scheduling this week, coming in next week for the test, and then waiting for the results. This whole process is genuinely cumbersome and time-consuming.”

#### 3.3.3 Complex screening processes

The demand for convenient services impacts lung cancer screening compliance. Cumbersome appointment procedures, lengthy wait times, and fragmented appointment processes directly reduce screening willingness.

N1: “If the screening process were more efficient-such as offering weekend screenings, quick online appointments, or completing the entire process at a health check-up center-more people might stick to regular screenings.”

N2: “I wish the process were simpler and more accessible-like getting a CT scan during a routine physical. Having to schedule in advance and sometimes reschedule feels bothersome and time-consuming. Unless there’s a real issue forcing me to screen, I’d rather skip it. If the wait is this long, I’d likely give up on the screening.”

N5: “Hospital procedures are complicated and take forever to schedule. Like, my mom went in, and the doctor told her to come back a week later. Maybe their hospital is too busy or overcrowded, but they said she had to wait a whole week for the test.”

#### 3.3.4 Negative psychological perceptions

Negative psychological perceptions significantly impact lung cancer screening compliance and represent one of the key factors hindering active participation in lung cancer screening. The primary factor is the phenomenon of “stigma associated with illness,” reflecting the cultural stigmatization of cancer-related topics.

N2: “Many people still feel quite uncomfortable discussing cancer. They turn pale at the mention of it and might avoid getting checked even when there’s no apparent issue. That’s probably the mindset.”

N8: “I know others who have gotten sick, but everyone avoids talking about it. We do not discuss cancer because most people are really uncomfortable with it.”

N11: “I heard friends’ parents had lung cancer, but they never mentioned screening. Many people just do not like talking about it-they choose to avoid the subject. Maybe Chinese people generally dislike discussing illness or misfortune; it’s considered unlucky.”

Secondly, a widespread “lucky-chance mentality” persists, reflecting the common misconception that “no symptoms mean no illness.”

N11: “The main thing is this mindset of ‘I’m fine.’ If I feel my body is healthy and problem-free, I probably will not get checked.”

N14: “Since I did not understand the importance of screening, I always thought, ‘No symptoms means no disease,’ so there was no need for a checkup.”

Finally, there’s the direct barrier of “medical aversion,” an instinctive avoidance of healthcare.

N3: “It’s my own mindset that resists it-no external factors, just my personal reluctance.”

N13: “Medical aversion-I’m quite averse to it myself.”

#### 3.3.5 Policy reference effects

Policy support and free screening programs significantly boost participation in lung cancer screening. Respondents consistently expressed strong expectations for government-led free screening initiatives.

N6: “Is this screening free? Like the cervical cancer screening-it would definitely be better if it were free.”

N7: “My job involves managing cervical and breast cancer screenings. Initially, uptake was low, but now rural residents are increasingly prioritizing their health. With the government offering various incentives, people are much more willing to participate.”

N11: “Free screenings would be ideal-many more would attend. Government-supported policies offer safety and assurance, so I believe everyone would actively participate. I believe cervical and breast cancer screenings for women are already free.”

## 4 Discussion

This study, grounded in the TPB framework, examined factors influencing lung cancer screening behavior among first-degree relatives of lung cancer patients. Findings indicate that screening behavior is jointly influenced by three core variables: behavioral attitude, subjective norm, and perceived behavioral control. Through an in-depth analysis of barriers, the study proposes a multidimensional intervention pathway to enhance screening behavior intention, specifically including: (1) Cognitive intervention: Strengthen health education to correct screening-related cognitive biases; (2) Implementation optimization: Streamline service processes to reduce barriers to screening participation; (3) Environmental support: Improve policy safeguards and foster a supportive social environment. Through these multi-level, coordinated strategies, first-degree relatives can be effectively encouraged to actively participate in lung cancer screening.

### 4.1 Strengthen health awareness education and correct screening cognitive biases

The findings of this study indicate that first-degree relatives of some lung cancer patients lack accurate understanding of lung cancer screening, exhibiting prevalent negative cognitive tendencies such as self-exclusion, complacency, and fear. These results align with the research by Quaife et al. (15). Carter et al.’s (18) qualitative study further highlights that even among high-risk groups who are long-term smokers and acknowledge the value of lung cancer screening, significant stigmatization and distrust toward medical care remain widespread.

To address these cognitive biases, systematic health literacy education is needed to correct misconceptions. Notably, clinicians play a crucial role in helping patients make screening decisions (19). This study found that first-degree relatives demonstrated high medical compliance during accompanying visits, exhibiting strong reliance on and willingness to follow treatment plans and health guidance provided by physicians. From a clinical practice perspective, this dependency likely stems from two sources: first, a natural inclination toward professional advice due to limited medical knowledge; second, the influence of China’s traditional medical culture characterized by a “physician-led, patient-cooperative” model. Therefore, it is recommended to enhance disease and screening knowledge education for first-degree relatives in clinical settings, establish multidisciplinary collaboration mechanisms, and integrate human-machine interaction technologies from artificial intelligence robots to develop a tiered education program for high-risk individuals with a family history of lung cancer. Specific measures could include: first, establishing a foundation of genetic risk awareness through interpretation of genetic test reports and family pedigree analysis. Subsequently, utilizing artificial intelligence robots to conduct interactive Q&A sessions and play animated short videos to clearly explain familial genetic risks and the importance of early screening, while using real-life cases to dispel the misconception that “no symptoms equals health.” In addition, future efforts should focus on establishing a family-oriented intervention model—that is, building a lung cancer screening promotion system centered on the family unit. Specifically, leveraging the critical moment of a lung cancer diagnosis—when the patient’s treatment plan is being finalized—systematic health education on lung cancer prevention and control could be provided to first-degree relatives who are accompanying or visiting the patient. By incorporating the concept of shared decision-making between doctors and patients, this approach would help first-degree relatives of lung cancer patients conduct personalized risk assessments based on their own smoking history, age, and family history, thereby enabling them to make informed decisions regarding screening. Finally, consolidate knowledge transfer through regular follow-ups at community health centers to enhance proactive screening compliance among high-risk individuals.

To address information gaps in rural areas, healthcare professionals can be organized to conduct regular outreach visits to villages for free medical consultations and educational sessions. Utilizing multiple channels such as village broadcasts and bulletin boards, they can disseminate screening knowledge to alleviate public fear of cancer, gradually eliminate cultural stigma surrounding the disease, and enhance public recognition of screening as a proactive preventive measure.

It is noteworthy that the average age of participants in this study was only 38.93 years, lower than the recommended starting age of 45 years for lung cancer screening. This age difference can easily lead to key cognitive biases, causing individuals to underestimate their own risk of developing the disease and subjectively believe that lung cancer screening is irrelevant to them. This cognitive bias of “young people have no risk” is a significant factor hindering high-risk individuals with a family history of lung cancer from proactively undergoing early screening. These results suggest that risk education and communication should focus on correcting the misconception that “family history only has screening value in advanced age.” Health education should emphasize that a family history of lung cancer is an independent high-risk factor independent of age.

Finally, some participants showed significant conceptual confusion regarding the distinction between genetic and environmental factors when trying to understand the familial clustering of lung cancer. One participant attributed his father’s lung cancer entirely to decades of smoking and lifestyle habits, while firmly denying the role of genetic factors. This cognitive tendency toward extreme dichotomization—attributing causation to either pure genetic determinism or pure environmental determinism—overlooks the complex patterns of gene–environment interactions involved in the development of lung cancer. The potential danger of this cognitive bias lies in the fact that if individuals believe they lack the same behavioral risk factors as their affected relatives (such as active smoking), they may mistakenly underestimate their independent risk of developing the disease due to their shared genetic background. Therefore, health education interventions should move beyond the cognitive framework of simple attribution and systematically elucidate the multifactorial nature of familial lung cancer risk, helping high-risk first-degree relatives develop a comprehensive risk perception model.

### 4.2 Optimize screening service processes and reduce implementation barriers

Based on participants’ descriptions of the screening service process, simplifying the service process is considered an effective way to reduce barriers. Existing screening services commonly suffer from long appointment waiting periods and cumbersome examination procedures, further increasing the practical difficulty of participation. This finding aligns with results from other screening studies (20). Additionally, screening times at most medical institutions overlap with individuals’ work schedules, and screening facilities are often located far from residential areas, creating significant time and transportation barriers (21). Cai et al.’s (22) implemented a “one-stop service” model for hepatitis C prevention and control, covering the entire process from screening and referral to treatment mobilization and follow-up. This approach effectively increased screening, treatment, and follow-up rates while reducing patient waiting times and dropout rates. Drawing from this experience, this study recommends implementing a “lung cancer screening service package” model. Specific measures include providing one-stop services at primary care facilities encompassing questionnaire-based initial screening, LDCT scans, and professional report interpretation; establishing an information-based intelligent appointment system with time-slot precision scheduling to reduce waiting times, along with offering evening and weekend screening clinics to accommodate work-constrained populations; Developing standardized screening protocols and integrating AI-assisted diagnostic systems to enhance primary-level screening quality; Establishing post-screening follow-up mechanisms, providing expedited referral pathways for positive cases, and launching a “post-screening psychological counseling hotline” to comprehensively reduce barriers throughout the screening process.

### 4.3 Strengthen policy and social support systems to foster a positive screening environment

First, the findings reveal that economic pressure constitutes a key barrier affecting individuals’ perceived behavioral control, directly constraining their screening decisions. High-income households are better equipped to bear screening-related medical, transportation, and time costs; conversely, low-income individuals, often burdened by livelihood pressures, tend to neglect preventive health investments, resulting in consistently lower screening participation rates. This finding aligns with other studies, where cost issues remain a core constraint in promoting LDCT screening across the United States-even in high-income countries, out-of-pocket expenses may impose financial burdens on low-income populations, leading to low screening utilization rates (23). It is worth noting that most respondents strongly expressed their hope for policy support, urging the government to implement public health programs similar to the “Two Cancers Screening” program for women. By lowering the financial barrier through free or reduced-cost services, participants believe this could help increase their willingness to undergo LDCT screening. China’s “Two Cancers” screening policy (for breast and cervical cancer) serves as a vital public health strategy for preventing these cancers, providing critical safeguards to increase screening coverage (22). Therefore, we recommend that the government draw upon the established operational model of the “Two Cancers” screening program. It should incorporate LDCT screening for high-risk groups (such as first-degree relatives of lung cancer patients) into key national public health service initiatives and develop medium-to-long-term plans. Specifically, efforts should be made to establish a unified national screening subsidy policy that provides targeted cost reductions for the target population. However, China’s basic medical insurance currently has significant limitations regarding routine outpatient coverage for LDCT lung cancer screening. In most regions, screening costs for asymptomatic high-risk populations have not yet been included in the universal reimbursement catalog, which to some extent reduces screening compliance among first-degree relatives. In response, this study recommends promoting the establishment of a unified national screening subsidy policy to provide targeted fee reductions for the target population and to include key screening procedures, such as LDCT, within the scope of medical insurance coverage, thereby substantially lowering the financial barrier. At the same time, commercial health insurance could be introduced as a supplementary payment channel to encourage families with the means to purchase specialized cancer screening insurance or critical illness riders, thereby covering the annual cost of LDCT examinations under commercial insurance claims. Through a multi-tiered payment model—where “basic medical insurance covers treatment costs and commercial insurance covers screening costs”—it is expected that the financial barrier to out-of-pocket screening for first-degree relatives will be lowered, thereby increasing their willingness to undergo screening. It is worth noting that when promoting this strategy in the future, attention must be paid to potential health equity issues arising from disparities in access to commercial insurance to prevent economically disadvantaged families from being further excluded from targeted prevention due to their inability to afford commercial insurance.

Second, this study also found that first-degree relatives of lung cancer patients exhibit high levels of trust and reliance on healthcare professionals’ recommendations, consistent with Draucker et al.’s findings (24). Therefore, healthcare providers should be empowered to strengthen their proactive intervention responsibilities at the clinical level. Given that healthcare professionals’ recommendations play a crucial “normative” role in individual screening decisions, the healthcare system should proactively enhance its intervention responsibilities in screening promotion to effectively implement opportunistic screening. We therefore recommend adding a “Lung Cancer Patient Relative Screening Management and Reminder” module to hospital information systems. Upon patient diagnosis, the system would automatically trigger alerts prompting physicians to enroll first-degree relatives into screening management cohorts. Doctors may provide family members with standardized, personalized screening recommendations that clearly explain their high-risk status and the necessity of screening, and may proactively refer first-degree relatives who meet the screening criteria for LDCT screening. Additionally, case managers or dedicated staff should conduct regular telephone follow-ups and provide enhanced guidance to family members who fail to undergo screening as scheduled. Through proactive intervention by the healthcare system, high-risk individuals can be ensured not to be overlooked, thereby transforming the patient’s “moment of care” into a “screening opportunity” for their family members.

Third, some participants from rural areas perceived relatively low health awareness in their regions, attributing this to limited access to health information and fewer health education and outreach activities, rather than a lack of individual capacity. This view aligns with structural barriers, such as insufficient resources in rural primary healthcare institutions and lagging health promotion efforts in rural areas. Actively integrating social resources and establishing community-based social support networks is crucial. A dual-track community mobilization strategy is recommended: administratively, establish a permanent “screening liaison” mechanism through community committees, with dedicated liaisons such as community doctors and Women’s Federation cadres conducting targeted screening awareness campaigns via regular home visits and neighborhood mutual aid initiatives. Systematically track and prioritize mobilization efforts for identified high-risk individuals. In addition, for rural areas with limited medical resources, the government could consider introducing mobile LDCT screening vehicles as a supplementary measure, enabling high-risk populations to receive screenings right in their communities and thereby addressing the shortfall in coverage by fixed medical facilities. Culturally, local volunteers such as village doctors and retired teachers can be selected and trained. Leveraging their dialect proficiency and relatable communication styles, they can deliver community health education that organically integrates screening knowledge into everyday life contexts. For example, home visits can be conducted for high-risk populations to assist them in scheduling screening appointments and guide them through the screening process, thereby reducing barriers to screening caused by unfamiliarity with the process. This approach aims to raise public awareness and acceptance of screening and promote its widespread adoption.

Finally, it is recommended that the government provide institutional support for LDCT screening at two levels of employment: first, by enacting specialized occupational health protection policies for high-risk occupational exposure groups; and second, by implementing flexible work arrangements and paid health leave for all workers to eliminate behavioral barriers—such as conflicts with work schedules—that lead to the abandonment or postponement of screening.

## 5 Limitations

This study has certain limitations. First, this study did not stratify participants by smoking status due to the small number of smokers in the sample (*n* = 3). We hope that future studies will further explore this issue using larger, more diverse samples. Second, the authors recruited participants exclusively from family members accompanying hospitalized patients at a single hospital, which introduces selection bias. The study’s findings may overestimate first-degree relatives’ awareness of and willingness to participate in screening. These participants may exhibit higher levels of health engagement, a stronger sense of family responsibility, or a more positive attitude toward the healthcare system. Future studies are advised to broaden their recruitment scope to include first-degree relatives in the community, those not accompanying patients to medical appointments, and those who are not direct caregivers. Thirdly, the study sample consisted primarily of urban participants, and the rural population was underrepresented. Fourth, all data were derived from participant self-reports and may be subject to social desirability bias and volunteer bias. Fifth, as an exploratory study based on single-interview data, this research did not employ triangulation methods for cross-validation, nor can it infer causal relationships or identify predictors between various factors and screening behavior. Sixth, this study was conducted within the context of China’s healthcare system and at a single hospital; therefore, caution should be exercised when applying its conclusions to other healthcare systems or cultural contexts. Seventh, as this study is based on participants’ self-reported perceptions and intentions, and only one participant had a history of actual screening, the results primarily reflect screening intentions rather than actual behavior; they should not be interpreted as a prediction of their actual behavior. Eighth, this study uses the TPB as an analytical framework, providing clear theoretical guidance for the systems analysis. However, the constructivist nature of this framework may lead to the neglect of some complex experiences that are difficult to categorize and make it difficult to fully present the cross-functional mechanisms of emotions, culture, institutions, and family relationships behind screening behaviors. Future research should expand sample sources to include participants from different regions and healthcare facilities of varying levels to enhance the generalizability of findings. Additionally, subsequent studies are recommended to adopt a mixed-methods approach, combining quantitative analysis with qualitative data to explore more comprehensively and deeply the various factors influencing screening decision-making behaviors among first-degree relatives of lung cancer patients.

## 6 Conclusion

This study systematically examined the factors influencing lung cancer screening behavior among first-degree relatives of lung cancer patients using a qualitative research approach grounded in the TPB framework. Findings indicate that screening behavior is primarily shaped by three interrelated factors: behavioral attitudes (widespread recognition of screening value despite cognitive biases), subjective norms (critical roles of family support and physician recommendations), and perceived behavioral control (financial constraints, time limitations, procedural complexity, and policy support levels). Based on these findings, healthcare providers are advised to strengthen health education tailored to first-degree relatives’ needs to correct misconceptions, optimize screening service processes to enhance accessibility and advocate for improved policy support to alleviate financial burdens. These measures will effectively reduce multiple barriers to screening participation, thereby comprehensively improving screening rates, treatment adherence, and follow-up completion rates. This study provides a theoretical foundation for developing targeted intervention strategies for high-risk populations. Future efforts should focus on constructing systematic lung cancer screening promotion programs and validating their practical effectiveness through large-scale intervention studies.

## Data Availability

The raw data supporting the conclusions of this article will be made available by the authors, without undue reservation.
