# Supplementary material for: Influencing factors of low-dose computed tomography screening behavior among first-degree relatives of lung cancer patients based on the theory of planned behavior: a qualitative study
Source: Front Public Health. 2026 Aug 21;14:1896783. doi: 10.3389/fpubh.2026.1896783 (PMC13543869; doi:10.3389/fpubh.2026.1896783)
Supplement: Supplementary file 1 [file Table_1.docx]

Influencing factors of Low-dose computed tomography screening behavior among first-degree relatives of lung cancer patients based on the theory of planned behavior: A qualitative study

| **Number** | **Item** | **Guiding questions** | **Explanations** |
| --- | --- | --- | --- |
| **Area 1: Research team and reflexivity** | | | |
| Personal characteristics | | | |
| 1 | Interviewer/facilitator | Which author/authors conducted the interview or focus group? | The first author conducted the interview. |
| 2 | Identity information | What were the credentials of the researcher? | Yaming He and Xiaokun Wu hold a master's degree in nursing, and the remaining authors hold a bachelor's degree in nursing. |
| 3 | Profession | What was their occupation at the time of the study? | All authors are nurses. |
| 4 | Gender | Was the researcher a man or a woman? | All authors are women. |
| 5 | Experience and training | What experience or training did the researcher have? | All authors have completed qualitative research courses, possesses extensive experience in qualitative research. |
| Relationship with participants | | | |
| 6 | Relationship status | Was a relationship established before the training started? | Build good relationships before research begins. |
| 7 | Participant's interviewer information | What did participants know about the researcher? | The participants know that the researchers are nurses from the department. |
| 8 | Interviewer characteristics | What characteristics were reported about the interviewer/facilitator? | At the beginning of each interview, participants were informed about the aim and objectives of the study. |
| **Area 2. Study design** | | | |
| Theoretical framework | | | |
| 9 | Methodological Orientation and Theory | Which methodological orientation was specified to support the study, e.g.,ethnography, phenomenology, content analysis? | This was a phenomenological approach. |
| Participant selection | | | |
| 10 | Sampling | How were participants selected? e.g., purposeful, convenience, consecutive, snowball | Criterion sampling method, one of the purposive sampling methods, was used. |
| 11 | Approach method | How were participants approached? For example, face-to-face, telephone, mail | Face-to-face interviews were conducted with the patients before starting the study. The timing of the interviews was determined by the patients who voluntarily agreed to participate in the study. |
| 12 | Sample size | How many participants were there in the study? | A total of 14 patients were included in the study. |
| 13 | Disagree | How many people refused to participate or dropped out? | No patient refused to participate in the study. |
| Setting | | | |
| 14 | The setting of data collection | Where was the data collected?E.g., home, clinic, workplace | Detailed information is provided in the data collection section of the study. |
| 15 | Presence of non-participants | Was there anyone else present apart from the participants and the researchers? | No other personnel was present. |
| 16 | Description of the sample | What are the essential characteristics of the sample? | First-degree relatives of lung cancer patients. |
| Data collection | | | |
| 17 | Interview guide | Did the authors provide questions, prompts, and guidelines? Has it been pilot-tested? | Detailed information was given in the Methods section. |
| 18 | Repeat interviews | Have there been re-interviews? If yes, how many? | No. |
| 19 | Audio/visual recording | Was audio or visual recording used to collect data in the study? | The interviews were recorded using a voice recorder. |
| 20 | Field notes | Were field notes taken during and/or after the interview or focus group? | All patients' responses and researcher observations were recorded. |
| 21 | Duration | How long were the interviews or focus groups? | Each interview lasted between 21 and 33 min. |
| 22 | Data saturation | Has data saturation been discussed? | Data saturation was discussed. |
| 23 | Transcripts returned | Have transcripts been returned to participants for comments and/or corrections? | Yes. |
| **Area 3: Analysis and findings** | | | |
| 24 | Number of data coders | How many data coders coded the data? | Researcher 1 and 2 conducted the data coding. |
| 25 | Description of the coding tree | Did the authors describe the coding tree? | The titles and subtitles in the results section represent the final coding tree. |
| 26 | Derivation of themes | Were the themes predetermined or derived from the data? | Themes were derived from the data. |
| 27 | Software | What software, if any, was used to manage the data? | Interview transcripts were systematically analyzed using NVivo 15.0 software. |
| 28 | Participant control | Provide feedback on the findings. | No. |
| Reporting | | | |
| 29 | Quotes provided | Are participant quotes presented to illustrate themes/findings? Is each quote identified, e.g., participant number | Conclusion. Participant quotes are presented to illustrate themes/findings. |
| 30 | Data and findings consistent | Was there consistency between the data presented and the findings? | Yes. |
| 31 | Clarity of main themes | Are the main themes presented in the findings? | Yes. |
| 32 | Clarity of small themes | Is there an explanation of the different cases or a discussion of minor issues? | Yes. |
